# Supplementary material for: Identification of photoperiod-induced specific miRNAs in the adrenal glands of Sunite sheep (Ovis aries)
Source: Front Vet Sci. 2022 Jul 22;9:888207. doi: 10.3389/fvets.2022.888207 (PMC9354845; doi:10.3389/fvets.2022.888207)
Supplement: Supplementary file 1 [file Data_Sheet_1.ZIP › Supplementary file/miRDeep2 file.zip!/miRDeep2 file/Novel_Structure/SPLP42A3/Novel_256.pdf]

Provisional ID	:	NC_019458.2_1003
Score total	:	0.8
Score for star read(s)	:	-1.3
Score for read counts	:	0
Score for mfe	:	0.5
Score for randfold	:	1.6
Score for cons. seed	:	
Total read count	:	399
Mature read count	:	399
Loop read count	:	0
Star read count	:	0

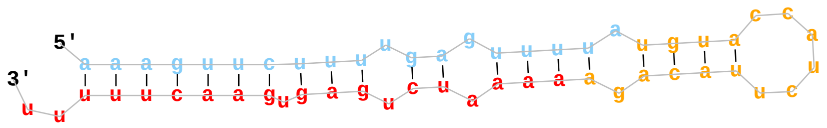

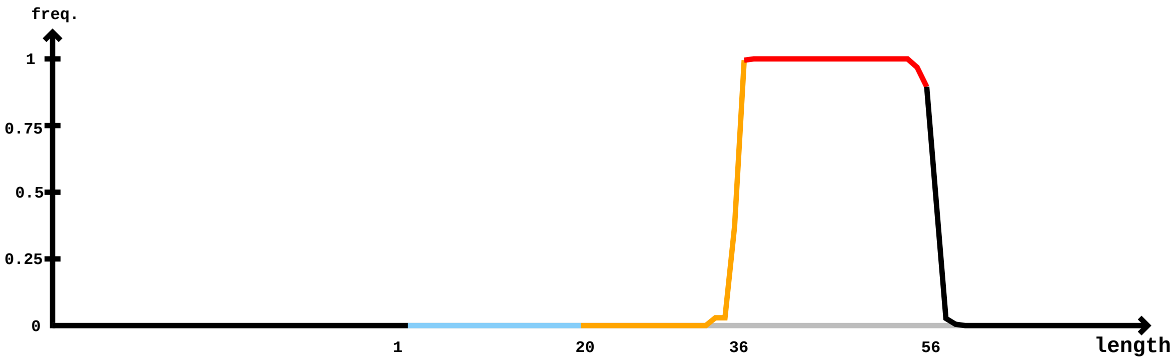

Star Mature

```
5'- auauuuuuauuuguaacuaaguaugggcugaaaaaaaaguucuuuugaguuuuauguaccaucuuacagaaaaaucugagugaacuuuuuggccagcccaguauuaaauaca  -3'   exp
    ..........((((..(((...(((((((...(((((((((((.((.((((.((((......)))).)))).)).))).)))))))))...)))))))...)))..))))   reads    mm     sample
    ...................................................................agaaaaaucugaAugaacuuu........................   1        1      seq
    ...................................................................agaaaaaucugaAugaacuuuu.......................   3        1      seq
    ...................................................................agaaaaaucugaAugaacuuuuu......................   8        1      seq
    .....................................................................aaaaaucugaAugaacuuu........................   12       1      seq
    .....................................................................aaaaaucugagugaacuuuu.......................   1        0      seq
    .....................................................................aaaaaucugaAugaacuuuu.......................   26       1      seq
    .....................................................................aaaaaucugaAugaacuuuuu......................   78       1      seq
    .....................................................................aaaaaucugaAugaacuuuuug.....................   24       1      seq
    ......................................................................aaaaucugaAugaacuuuuu......................   247      1      seq
    ......................................................................aaaaucugagugaacuuuuugg....................   9        0      seq
    .......................................................................aaaucugagugaacuuuuuggc...................   2        0      seq
```
